# Evaluating SH-SY5Y cells as a dopaminergic neuronal model: morphological, transcriptomic, and proteomic insights

**DOI:** 10.55730/1300-0152.2772

**Published:** 2025-08-11

**Authors:** Eylül Ece İŞLEK CAMADAN, Mehmet SARIHAN, Murat KASAP, Gürler AKPINAR, Elifcan KOÇYİĞİT

**Affiliations:** Proteomics Laboratory, Department of Medical Biology, Faculty of Medicine, Kocaeli University, Kocaeli, Turkiye

**Keywords:** Neurogenic differentiation, SH-SY5Y cells, dopaminergic markers, Parkinsons Disease model

## Abstract

**Background/aim:**

The SH-SY5Y neuroblastoma cell line is a popular in vitro model for neurodegenerative disease research, especially Parkinsons disease (PD) research, but its use is complicated by limitations like the persistence of neuroblastoma-like features, unstable differentiation, mitochondrial dysfunction, and cellular stress. To address these limitations, this study tested a blended, nine-day differentiation protocol that sequentially applied all-trans retinoic acid (RA), brain-derived neurotrophic factor (BDNF), and dibutyryl cyclic adenosine monophosphate (dbcAMP). By evaluating key neuronal, dopaminergic, and PD-related markers, the research aims to determine if these differentiated SH-SY5Y cells are a suitable model for studying PD.

**Materials and methods:**

A blended differentiation protocol using RA, BDNF, and dbcAMP was applied to SH-SY5Y cells. Morphological changes were evaluated by immunofluorescence microscopy. Furthermore, mostly dopaminergic neuronal markers associated with PD were used for characterization purposes. Nanoliquid chromatography coupled with tandem mass spectrometry proteome analysis was performed to identify changes in protein expression related to differentiation.

**Results:**

Differentiation led to neuron-like morphology with extended neurites. Gene expression analyses revealed upregulation of several neuronal markers, such as Nestin and MAP2, indicating progression from progenitor to neuron-like states. Furthermore, some dopaminergic markers, such as TH and Nurr1, showed elevated expression with asynchronous expression patterns, suggesting heterogeneity in the differentiation process. Proteomic analysis indicated significant changes in cell differentiation and neurogenesis. Transient expression of key neuronal markers was observed. The cells required continuous external stimuli.

**Conclusion:**

While SH-SY5Y cells exhibited dopaminergic characteristics following the blended differentiation protocol, the transient expression of key neuronal markers and the need for continuous external stimuli raised concerns about the stability and functional maturity of these differentiated cells as an in vitro PD model. These findings suggest that SH-SY5Y cells might not fully capture the properties of mature neurons.

## 1. Introduction

One of the most frequently used immortal cell lines that has been used as a model in neuronal degeneration studies since the early 1980s is the SH-SY5Y neuroblastoma cell line (Påhlman et al., 1984). The significance of these cells comes from the fact that they present a catecholaminergic phenotype and harbor pathways that can synthesize dopamine and noradrenaline (Ioghen et al., 2023). They also display a moderate level of dopamine-β-hydroxylase activity and have negligible levels of choline acetyl-transferase, acetyl-cholinesterase, and butyryl-cholinesterase activities (Xicoy et al., 2017; Hoffmann et al., 2023). SH-SY5Y cells also show noradrenaline release and tyrosine hydroxylase (TH) activity at basal levels (Nagatsu et al., 1964; Ross and Biedler, 1985). In addition, a low level of sodium-dependent dopamine transporter (DAT) expression, which is expressed only in dopaminergic neurons in the central nervous system, is observed in these cells. Many genes and pathways related to the pathogenesis of PD are claimed to be intact in SH-SY5Y cells (Krishna et al., 2014). Although all these features are not sufficient to classify SH-SY5Y cells as dopaminergic neuron-like cells, it is possible to find many studies in the literature in which these cells are used as a model in PD (Kasap et al., 2017; Ioghen et al., 2023).

In their undifferentiated form, SH-SY5Y cells appear to represent a cancerous rather than a neuronal phenotype (Şahin et al., 2021). To transform these cells into a neuronal type, researchers subject them to neuronal differentiation. Apparently, the differences between the differentiated and undifferentiated cells are far more than the morphological changes that are observed. For example, the differentiated cells, similar to neurons, have a limited growth potential with a polarized morphological structure and can express some of the mature neuronal markers (Kovalevich and Langford, 2013; Shipley et al., 2016; Hromadkova et al., 2020). Like mature dopaminergic neurons, the cells can also display increases in adenosine triphosphate (ATP) levels and plasma membrane activities in addition to a decrease in mitochondrial membrane potential. While SH-SY5Y cells are a common PD model, using undifferentiated cells presents several disadvantages, which are continuous proliferation, lack of mature neuronal markers, limited catecholamine synthesis, and sensitivity to neurotoxins seen in primary neurons (Xie et al., 2010). However, despite their common use and their certain neuron-like characteristics, it is still not clear whether SH-SY5Y cells represent a true in vitro dopaminergic neuron-like cell type in their differentiated form (Lopes et al., 2017).

We know that the differentiation of SH-SY5Y cells has been tested in several studies to generate adrenergic, cholinergic, or dopaminergic neuron-like cells. In those studies, various differentiation reagents were used individually or in combination. Some of those reagents are 12-o-tetradecanoyl phorbol-13-acetate (TPA) (Alrashidi et al., 2021), dibutyryl cyclic adenosine monophosphate (dbcAMP) (Bell and Zempel, 2021), all-trans retinoic acid (RA) (Avola et al., 2018), brain-derived neurotrophic factor (BDNF) (Hromadkova et al., 2020), nerve growth factor (NGF) (Hartman and Hertel, 1994), cholesterol (Teppola et al., 2016), and vanadate (Bayeva et al., 2021). Each of these reagents acts on different metabolic pathways at different levels. Moreover, the final phenotype may differ depending on the treatment with differentiation agents alone or in combination. Leveraging the literature, this study developed a shortened and highly effective neurogenic differentiation protocol by employing a sequential treatment strategy using RA, BDNF, and dbcAMP reagents to initiate, advance, and maintain the neurogenic differentiation process. The mechanism of action of RA is the stimulation of the expression of dopaminergic receptors. As cells are exposed to RA, their axon length increases. Furthermore, the synthesis of neuron-specific enzymes such as acetylcholinesterase and neurotransmitters (e.g., catecholamines like dopamine) increases, while cytoskeletal changes (e.g., neurofilaments) and electrophysiological modifications, as observed in normal neurons, are noted (Melino et al., 1997). RA treatment was supposed to induce expression of cell surface tropomyosin receptor kinase (TrkB) receptors, making cells responsive to BDNF (Kaplan et al., 1993), while BDNF in turn should have activated phosphatidylinositol 3-kinase (PI3-K) and extracellular regulated kinase (ERK) pathways to mediate cell survival and neurogenesis (Encinas et al., 1999). SH-SY5Y cells subjected to neuronal differentiation protocols with RA and BDNF, respectively, exhibit neuron-like structures and axon extensions rather than their round morphologies (Encinas et al., 2000; Targett et al., 2024). Furthermore, dbcAMP encourages neuronal differentiation by affecting the cAMP-dependent protein kinase A (PKA) signaling pathway. dbcAMP promotes differentiation into an adrenergic phenotype, leading to increased noradrenaline production and enhanced TH expression (Kume et al., 2008).

Herein, we asked how appropriate it is to use these differentiated cells as a model in in vitro PD studies. The assessment of the differentiation process was monitored by following the changes in cell morphology and cellular growth rate, as well as changes in mRNA and protein levels of mature and dopaminergic neuronal markers and PD-associated markers. Based on the literature, in this study, the neurogenic markers such as Nestin (NES), neuronal differentiation 1 (NeuroD1), neuronal nuclei protein (NeuN, microtubule associated protein 2 (MAP2), and nuclear receptor subfamily 4 group a member 2 (Nurr1); dopaminergic pathway markers such as TH, DAT, dopa decarboxylase (DDC) and dopamine receptor D1 (DRD1); PD related markers such as PTEN induced kinase 1 (PINK1), parkin RBR E3 ubiquitin protein ligase (PARK2), parkinsonism associated deglycase (DJ-1), and VPS35 retromer complex component (VPS35) were chosen for characterization (Murillo et al., 2017; Scott et al., 2017; Lai et al., 2020; Alaylıoğlu et al., 2024). Due to its high sensitivity, specificity, and ability to accurately quantify low concentrations of analytes in complex biological samples, nanoliquid chromatography coupled with tandem mass spectrometry (nLC-MS/MS) proteome analysis has also been performed to monitor changes occurring at the protein level on a global scale.

## 2. Materials and methods

A list of all chemicals used in this study is available in Supplementary File 1.

### 2.1. Cell culture

SH-SY5Y cells were purchased from the American Type Culture Collection (ATCC) and maintained in Dulbeccos modified Eagles medium (DMEM) supplemented with 10% heat-inactivated fetal bovine serum (hiFBS) and incubated in a humidified incubator with 5% CO_2_ at 37 C (Shipley et al., 2016). When the cells reached 70%–80% confluence, they were split into T-75 (15**–**20 mL) culture flasks for initiation of neuronal differentiation.

### 2.2. Neuronal differentiation of SH-SY5Y cells

A modified sequential differentiation protocol was developed based on the literature (Encinas et al., 2000; Kovalevich and Langford, 2013; Forster et al., 2016; Shipley et al., 2016). DMEM without sodium pyruvate (DMEM w/o SP) was used during the differentiation process (Forster et al., 2016). All media used in this protocol were prepared in 5-mL volumes. Cells were maintained in a 35-mm^2^ plate (5 mL) with basic growth medium composed of DMEM w/o SP supplemented with 5% hiFBS, 1× penicillin/streptomycin (P/S), and 2 mM L-glutamine. When they reached 70%–80% confluency, the basic growth medium was replaced with differentiation medium 1 (DM1), which was DMEM w/o SP supplemented with 2.5% FBS, 1× P/S, 2 mM L-glutamine, and 10 μM RA, and the day was marked as day 1. After 2 days of incubation, at day 3, the medium was replaced with differentiation medium 2 (DM2), which was DMEM w/o SP supplemented with 1% FBS, 1× P/S, 2 mM L-glutamine, and 10 μM RA. Cells were incubated in this medium for 2 more days and were transferred into MaxGel extracellular matrix (ECM)-coated plates and incubated in fresh DM2. On the ECM-coated plates, the cells were incubated for 2 more days, and the medium was replaced with neurobasal medium (NM) containing 1× B-27 supplement, 20 mM potassium chloride (KCl), 2 mM GlutaMAX supplement, 50 ng/mL BDNF, 1× P/S, and 2 mM dbCAMP at day 7. After two more days of incubation, at day 9, cells were examined for neuronal differentiation under an inverted microscope. In this experimental setup, in parallel, undifferentiated SH-SY5Y cells were used as a control group, which were also grown in basic growth medium. Therefore, SH-SY5Y cells were cultured and routinely split as usual throughout the differentiation process.

### 2.3. Monitoring neuronal differentiation by immunofluorescence microscopy (IF) microscopy

To assess neural differentiation, the expression of selected neuronal differentiation markers Nestin, NeuN, NeuroD1, and Nurr1 was monitored using IF microscopy. Proper dilutions of each related antibody were made based on the recommendations by the manufacturers (Supplementary File 2). SH-SY5Y cells were cultured and differentiated in 35-mm^2^ plates with glass coverslips (Corning, US). On the other hand, undifferentiated SH-SY5Y cells were cultured and used as a control group. To ensure the reproducibility and statistical robustness of the findings, each antibody-specific IF experiment was performed with a minimum of three independent replicates with coverslips according to Kara et al. (2022). Cell nuclei were stained with 4′,6-diamidino-2-phenylindole (DAPI; 1 μg/mL) for 5 min according to the manufacturers recommendation. Imaging was performed with an inverted microscope (Olympus CKX41; Olympus, JP) equipped with an Olympus DP74 digital camera system with appropriate filters according to the wavelength scale of the secondary antibodies. The images were processed using ImageJ software (version 1.54 g).

### 2.4. Quantitative real-time PCR analysis of neuronal differentiation markers

For quantitative real-time polymerase chain reaction (RT-qPCR) analysis, ribonucleic acid (RNA) was independently isolated from two biological replicates using the RNeasy Mini Kit (Qiagen, US). Equal amounts of RNA from each replicate were pooled for complementary deoxyribonucleic acid (cDNA) synthesis, performed with the RevertAid First Strand cDNA Synthesis Kit (Thermo Fisher Scientific, US). In at least three independent experiments, qPCR was performed on the Roche LightCycler 480 II system (Roche, US) using SYBR Green chemistry for day 0, 3, 6, and 9 samples arranged as undifferentiated, midpoint 1, midpoint 2, and differentiated cells, respectively. Glyceraldehyde-3-Phosphate Dehydrogenase (*GAPDH*) was used as a housekeeping gene for normalization.

Expression levels of *NES*, *NeuroD1*, *NeuN*, *MAP2*, *Nurr1*, *TH*, *DAT*, *DDC*, *DRD1*, *PINK1*, *PARK2*, *DJ-1*, *VPS35*, *ACTB*, and *GAPDH* were monitored using optimized primer pairs from Qiagen SABiosciences (Qiagen, US; Supplementary File 2). The method used in these sections is basically known and used in the literature (Forster et al., 2016). Analysis of the raw data was carried out by LightCycler 480 Software, Version 1.5 (Roche, US). Relative gene expression was calculated using the 2^–ΔΔCT^ method, normalizing target cycle threshold (CT) values to a reference gene and control sample.

### 2.5. Agarose gel electrophoresis

A 2% agarose gel with ethidium bromide was used to visualize DNA products. The gel was visualized under ultraviolet (UV) light using a transilluminator (WUV-l50, DaiHan Scientific, SK). DNA bands were compared to a 50-bp DNA ladder for confirming the expected amplicon sizes, and images of the gel were captured for documentation (Lee et al., 2012).

### 2.6. Preparation of protein extracts

**Figure 1:**
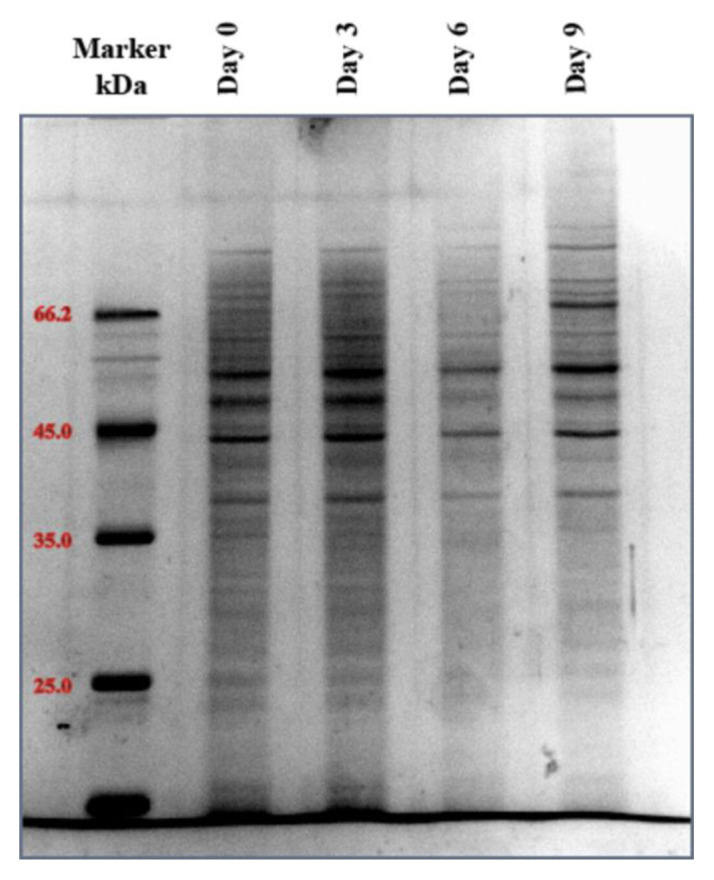
Protein profiling and validation by SDS-PAGE.

The protein extraction performed is well-established in the literature (Akpinar et al., 2017). Protein concentrations were determined by modified Bradford assay (BioRad, US), and protein quality was verified by sodium dodecyl sulfate polyacrylamide gel electrophoresis (SDS-PAGE) analysis (Figure 1). Protein extracts were then aliquoted, snap-frozen in liquid nitrogen, and stored at −80 C.

### 2.7. In-solution trypsin digestion

In-solution tryptic digestion was performed according to the manufacturers instructions using the In-solution Tryptic Digestion and Guanidination Kit (Thermo Fisher Scientific, US). A total of 20 μg of protein was digested as described (Yılmaz Tuğan et al., 2024), and the digestion solution containing peptides was vacuum-concentrated to dryness. The peptides were resuspended in 0.1% formic acid for analysis, and the peptide concentrations of the samples were measured using the Qubit assay (Invitrogen, US).

### 2.8. nLC-MS/MS analysis

The peptides were analyzed by nLC-MS/MS using an UltiMate 3000 RSLCnano System (Dionex, Thermo Scientific, US) coupled to a Q Exactive mass spectrometer (Thermo Scientific, US). Three independent runs were performed for each group. The peptide separation was achieved as described (Sarıhan et al., 2025). The applied gradient for separation was 6% in 5 min, 6%–20% B in 45 min, 20%–40% B in 30 min, 40%–90% B in 20 min, 90% in 20 min, 90%–6% B in 5 min, and 6% B for 5 min with the flow rate of 300 nL/min in a 130-min total run time. Full scan MS spectra were acquired with the following parameters: resolution 70,000, scan range 400–2000 m/z, target automatic gain control (AGC) 3 × 106, maximum injection time 60 ms with selecting the top 10 precursor ions.

### 2.9. Analysis of mass spectrometry data

The data collected were analyzed using the parameters presented in Sarihan et al. (2024) with Proteome Discoverer 2.2 software (Thermo Scientific, US). The human reference proteome databank ( UP000005640 ) was used for label-free quantification analyses (LFQ) of proteins.

### 2.10. Bioinformatics analysis

A heatmap clustering analysis was conducted for the protein profiling^1^. During the heatmap analyses, the average linkage was used as a clustering method, and the Euclidean algorithm was employed for the distance measurement method. To identify regulated proteins associated with cell differentiation (GO:0030154) and neurogenesis (GO:0022008), proteins with a p-value of <0.05 and fold change of >2 were analyzed. A comprehensive list of proteins involved in these biological processes was retrieved from the Gene Ontology (GO) knowledgebase, using the latest version^2^ and filtered to include only entries specific to *Homo sapiens* (TaxonID: 9606). This list was then compared with the set of regulated proteins obtained from UniProt,^3^ and filtering was applied to identify overlapping entries. As a result, proteins associated with the selected biological processes were identified and subsequently categorized. Additionally, pathway and protein–protein interaction analyses of the regulated proteins were conducted using the Search Tool for the Retrieval of Interacting Genes/Proteins (STRING) tool^4^. A False Discovery Rate threshold of < 0.01 was applied to identify significantly enriched biological processes and interaction networks. Finally, brain-specific proteins from the Human Protein Atlas^5^ were compared with those identified in this study.

### 2.11. Statistical analysis

The statistical analyses were performed using IBM SPSS software version 20.0 (IBM Co., Armonk, NY, US). The Kolmogorov–Smirnov test, as well as skewness and kurtosis values, were used to assess the normality of data distributions (George and Mallery, 2010). The data are expressed as mean ± standard deviation. A p-value of <0.05 was considered statistically significant. The RT-qPCR data were analyzed using the Qiagen GeneGlobe Data Analysis Center.^6^

## 3. Results

### 3.1. Morphological changes of the cells during differentiation

**Figure 2:**
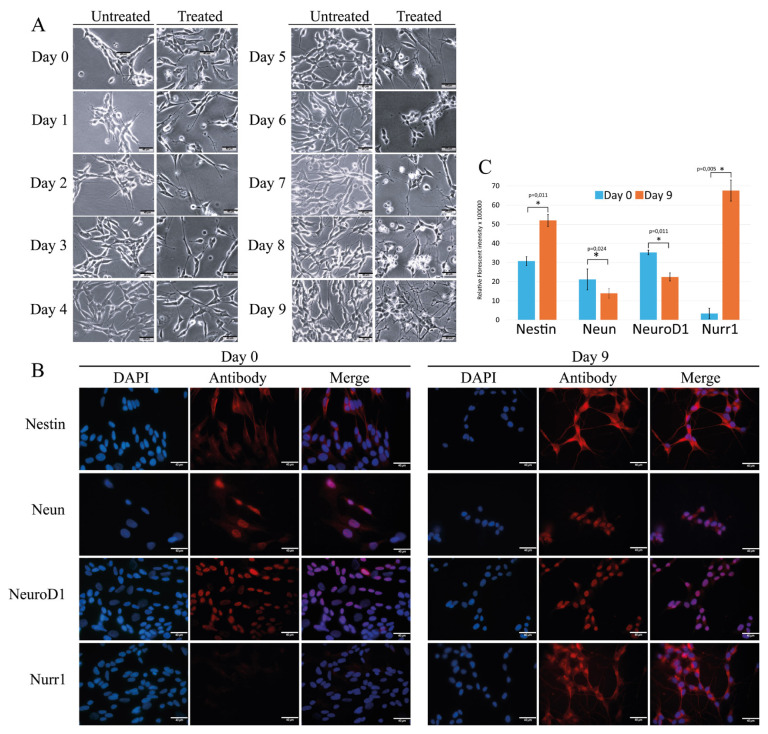
Representation of the differentiation processes of SH-SY5Y cell lines. (A) Phase-contrast microscopy images (40×) of SH-SY5Y cells demonstrating morphological changes during neurogenic differentiation. (B) IF analyses of SH-SY5Y cells for monitoring the changes in expression levels of selected neurogenic differentiation markers before (day 0) and after (day 9) differentiation. Cell nuclei were stained with DAPI, while the target antigens were visualized using Texas Red conjugated secondary antibodies. (C) Graphical presentation of IF showing the signal intensities of neural progenitor and neuronal markers Nestin, NeuroD1, Nurr1, and NeuN between day 0 and day 9. Statistical significance is indicated by asterisks (*p < 0.05).

The process of SH-SY5Y differentiation is presented in Figure 2A. In their undifferentiated form (day 0), SH-SY5Y cells displayed neuroblast-like, nonpolarized cell bodies with few truncated processes. They tended to grow in clusters and formed occasional clumps as cells appeared to grow on top of each other. They also displayed roundish features with short processes. The undifferentiated cells were subjected to a stepwise differentiation process. After 3 days of incubation, differentiation became apparent as cells displayed a neuron-like phenotype with loss of the round morphology. On day 9, a more mature neuron-like morphology with neurite extensions that often connected the cells was evident (Figure 2A).

### 3.2. Monitoring Nestin, NeuN, NeuroD1, and Nurr1 expressions by IF

IF experiments were performed on days 0 and 9 for each protein (Figures 2B and 2C). On day 0, the cells expressed Nestin, NeuN, and NeuroD1. Nurr1 expression was not at a detectable level in undifferentiated SH-SY5Y cells. On day 9, all four neuronal differentiation markers were expressed by the cells. The most pronounced increase in signal intensity was observed in Nurr1 expression. Like Nurr1, increases in signal intensities of Nestin, NeuN, and NeuroD1 were also detected in differentiated cells. The IF analyses showed that the expression levels of Nestin and Nurr1 were increased on day 9, whereas the levels of NeuN and NeuroD1 were significantly decreased.

### 3.3. Monitoring differentiation at the mRNA level by RT-qPCR

**Figure 3:**
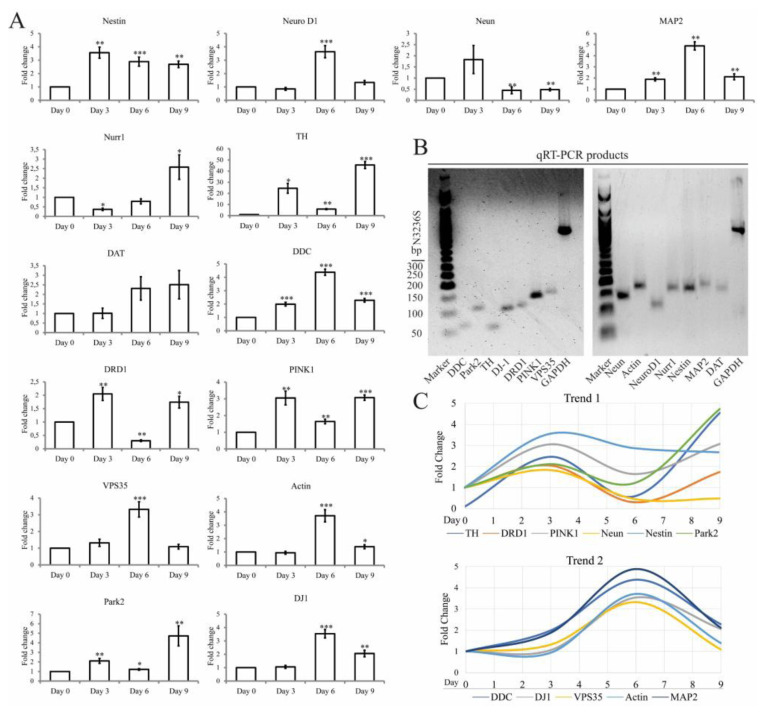
Monitoring differentiation at the mRNA level of target genes by qRT-PCR during the differentiation process of SH-SY5Y cells. (A) Determination of mRNA levels for selected neurogenic markers using RT-qPCR. (B) Images of RT-qPCR products used to quantify mRNA levels of selected neurogenic markers. (C) Temporal expression patterns of mRNA involved in neurogenesis. Trend 1 shows fold change in the expression of proteins involved in neurogenesis and dopaminergic neuron differentiation: TH, DRD1, PINK1, NeuN, Nestin, and Park2. These proteins exhibit varying expression dynamics, with TH and PARK2 showing an increase towards day 9, while DRD1 and NeuN reach a peak around day 6. Trend 2 highlights proteins related to neuronal function and maintenance. These proteins generally peak around day 6, followed by a gradual decline by day 9. Note that TH ratios were divided by 10 to fit the curve with others. Statistical significance was denoted as follows: p < 0.05 (*), p < 0.01 (**), and p < 0.001. (***).

RT-qPCR monitored changes in neurogenic differentiation marker expression on days 0, 3, 6, and 9 (Figure 3A). The final RT-qPCR amplification products were analyzed. All amplification products displayed single bands at the expected sizes (Figure 3B). According to the results, gene expression patterns during neurogenic differentiation reveal two distinct regulatory trends. Trend 1 included *TH*, *DRD1*, *PINK1*, *NeuN*, *NES* and *PARK2*, and Trend 2 comprised *DDC*, *DJ1*, *VPS35*, *Actin*, and *MAP2* (Figure 3C).

The gene expression patterns varied for each gene. *NES* expression was detected throughout the differentiation process. The lowest level of expression was observed at day 0. At day 3, a 3.5-fold increase in *NES* expression was detected. This expression level was maintained to a certain degree throughout differentiation. A 3.6-fold increase was detected in *NeuroD1* expression at day 6. This increase was followed by a drop to its previous level on day 9. There was a low level of *NeuN* expression in undifferentiated SH-SY5Y cells. The expression of *MAP2* gradually increased during neuronal differentiation and reached a maximum at day 6 with a 4.8-fold upregulation. However, on day 9, *MAP2* expression decreased to a level observed on day 3. To assess if the differentiation process produced dopaminergic neuron-like cells, we monitored the changes in expression levels of *Nurr1*, *TH*, *DAT*, *DDC*, and *DRD1*. The differentiated cells displayed increased expression of all dopaminergic neuronal markers. For some markers, the highest expression level was observed on days 3 or 6, and for others, the increase was observed on day 9 (Figure 3A).

Changes in expression levels of *PARK2*, *DJ-1*, and *VPS35* were monitored during differentiation since mutations in these proteins are associated with PD (Naren et al., 2023). Similar to neurogenic differentiation markers, differentiated cells displayed increased expression levels of *PARK2* (4.7-fold) and *DJ-1* (2.0-fold) at day 9. Both *DJ-1* and *VPS35* levels have also significantly increased at day 6.

### 3.4. Monitoring changes occurring at the proteome level during differentiation

To study changes occurring at the proteome level throughout differentiation, we performed nHPLC-MS/MS analysis. A total of 428 proteins were identified with high confidence (Supplementary File 3). A total of 108, 174, and 136 proteins were differentially regulated in samples prepared from day 3, day 6, and day 9, respectively, when compared with day 0. Of these regulated proteins, 71 were consistently regulated in all three time points. There were other proteins regulated differentially in a time point-dependent manner. There were also proteins shared between day 3 and day 6, and day 6 and day 9 (22 and 26 proteins, respectively).

**Figure 4:**
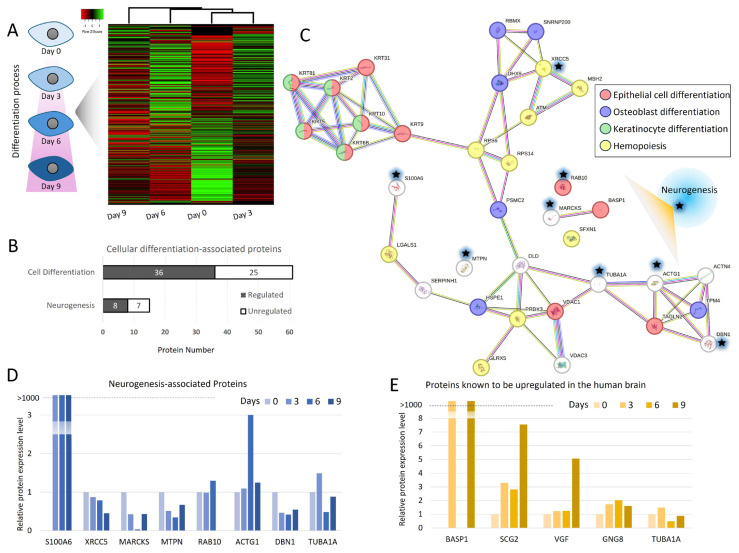
Bioinformatics analysis of proteins identified by nLC-MS/MS analysis. (A) The heatmap of regulated proteins between the groups showed hierarchical clustering of protein profiles. (B) A bar graph showing the number of proteins found to be associated with cellular differentiation processes and neurogenesis. (C) STRING analysis illustrating the protein-protein interaction networks of regulated proteins linked to differentiation. Proteins marked with a star (*) were found to be associated with neurogenesis. (D) Regulation trends of the proteins marked with stars on the STRING analysis that were found to be associated with neurogenesis. (E) Regulation trends of the proteins identified in this study were known to be upregulated in the human brain.

The molecular changes and network interactions associated with SH-SY5Y cell differentiation are presented in Figure 4. A heatmap analysis revealed that the differentiation process had a significant impact on the cellular proteome (Figure 4A). While the proteome profiles of pretreatment day 0 cells and cells on day 3 were relatively similar, the degree of similarity decreased noticeably on days 6 and 9. The highest degree of change was observed on day 9. Among the proteins associated with cellular differentiation, 36 showed increased expression and 25 showed decreased expression. Regarding neurogenesis, eight proteins were found to be upregulated, whereas the levels of seven proteins were downregulated (Figure 4B). A STRING analysis showed that the regulated proteins tend to promote epithelial, osteoblast, and keratinocyte differentiation (Figure 4C). In addition, nine of these proteins are directly linked to neuronal cell differentiation (Figures 4C and 4D). However, the proteins involved in these processes did not consistently increase; their expression levels fluctuated (Figure 4D). The S100A6 protein showed the most significant increase in expression. Additionally, five proteins specific to brain tissue were found to be regulated during the process. Among them, the expression levels of BASP1, SCG2, VGF, and GNG8 were significantly increased (Figure 4E).

## 4. Discussion

The use of the SH-SY5Y cell line as a neuronal cell model presents several significant limitations. These include the preservation of neuroblastoma-like characteristics during the differentiation, instability in differentiation fate, increased endoplasmic reticulum stress, and mitochondrial dysfunction (Hoffmann et al., 2023). Since this study focused on the suitability of using these differentiated cells as an in vitro model for PD research, the differentiation process was monitored by observing changes in cell morphology, growth rate, and the expression levels of key mRNA and proteins, including mature, dopaminergic neuronal, and PD-associated markers.

Initially, several challenges were encountered during the study, including inefficient attachment of cells to the ECM, a high rate of cell death, and resistance to neuronal differentiation. Additionally, the literature indicates that excessive cell passage can lead to replicative senescence, meaning that cells of the same quality cannot be obtained (Falkenburger and Schulz, 2006). Prolonged differentiation protocols also resulted in insufficient cell numbers for subsequent protein, IF, and RNA analyses. To address these issues, a comprehensive literature review was conducted, revealing that various studies achieved neuronal differentiation in shorter periods. Drawing upon these insights, an optimized nine-day differentiation protocol was developed, and the suitability of differentiated SH-SY5Y cells as a model for PD research was evaluated.

Preliminary trials demonstrated that this blended protocol effectively induced a high rate of morphological neuron-like differentiation in SH-SY5Y cells within 9 days, while also significantly reducing the mortality rate compared to previously tried methods. One of the highlights of the blended protocol was the use of DMEM w/o SP to increase the possibility of SH-SY5Y cells differentiating into N-type neuron-like cells rather than S-type epithelial-like cells. Furthermore, reducing the concentration of hiFBS between DM1 and DM2 during the differentiation process facilitated earlier cellular readiness for neuronal differentiation, typically prior to day 7. Subsequently, on day 7, cells were cultured in an entirely FBS-free medium. The lack of FBS in this medium led to a complete cessation of cell proliferation and the initiation of rapid differentiation. Since each medium triggers cellular differentiation differently, the differentiation process was monitored by dividing it into four checkpoints according to the medium change times.

The morphological changes observed during differentiation are consistent with previous studies where SH-SY5Y cells displayed neuroblast-like characteristics at early stages, transforming into neuron-like cells with long neurite extensions, indicating that the applied protocol successfully induced phenotypic changes commonly associated with mature neurons after differentiation (Encinas et al., 2000; Constantinescu et al., 2007). At the molecular level, the expression of key neuronal markers confirmed the neurogenic differentiation of SH-SY5Y cells. The transient upregulation of Nestin, a marker for neuronal progenitor cells (Bernal and Arranz, 2018), suggested that some neural progenitor characteristics remain active throughout the differentiation process. NeuN is a pre-mRNA alternative splicing regulator protein that localizes to the nucleus and is used as a marker specific for neurons (Guselnikova and Korzhevskiy, 2015). IF studies of NeuN revealed an increased punctate nuclear distribution in differentiated cells. It was also detected in the cytoplasm, suggesting an elevated level of NeuN synthesis. According to the RT-qPCR experiments, the expression of *NeuN* by undifferentiated cells suggested that some cells may have already been committed to a neuronal lineage. It is also possible that *NeuN* was expressed at a basal level in undifferentiated cells because mRNA processing is required for the survival of the cells under regular circumstances. However, during the differentiation process, *NeuN* expression initially increased but subsequently decreased. This pattern suggested successful initiation of neuronal differentiation, followed by potential neuronal stress. The expression level of another neuronal marker, *MAP2*, a cytoskeletal protein (Soltani et al., 2005), decreased by day 9 to the level observed by RT-qPCR on day 3, suggesting that SH-SY5Y cells have undergone significant neuronal differentiation, as evidenced by the initial increase in *MAP2* expression. However, there was a subsequent decrease in *MAP2* levels. While the initial increases in these markers indicated successful neuronal differentiation, the subsequent decline suggested that these cells might not fully sustain their differentiation state over extended periods. This transient expression pattern could imply that SH-SY5Y cells might not represent a fully mature and stable neuronal model.

The dopaminergic differentiation markers, including *TH*, *DAT*, *Nurr1*, and *DRD1*, were upregulated, with *TH* showing the most significant increase. *Nurr1* is a transcription factor that is crucial for the development and maintenance of dopaminergic neurons (Jankovic et al., 2005). The presence of these neurons can be confirmed by several markers. Specifically, *TH* is an enzyme involved in dopamine synthesis, making it a key marker for these cells (Daubner et al., 2011). Similarly, *DAT*, which is responsible for the reuptake of dopamine from the synaptic cleft, is also specific to dopaminergic neurons (Vaughan and Foster, 2013). Finally, *DDR1* is a receptor for dopamine, indicating functional dopaminergic neurons (Bhatt et al., 2000). The increase in *TH* aligns with the goal of creating dopaminergic neuron-like cells. However, the asynchronous peaks in the expression of dopaminergic markers indicated a complex and dynamic differentiation process with asynchronous cell responses. This highlights the importance of a multifaceted approach to studying differentiation, including increased sampling, functional validation, and consideration of cellular heterogeneity that may arise from differentiation agents, culture conditions, and the differentiation process (Xicoy et al., 2017).

Our research also focused on *PARK2*, *DJ-1* and *VPS35* genes since SHSY-5Y cells are often used as a model in studies associated with PD (Ioghen et al., 2023). The substantial increase in *PARK2* expression levels by RT-qPCR suggested that as SH-SY5Y cells differentiate into a more neuron-like state, there is an elevated need for Parkin, possibly due to increased mitochondrial activity and the necessity for maintaining protein homeostasis. On the other hand, the early and sustained increase in *DJ-1* levels suggested that oxidative stress management and protein folding are crucial during the early stages of neuronal differentiation and continue to be important as differentiation progresses. The significant increase at day 6 but not at day 9 in *VPS35* levels may indicate a transient need for enhanced endosomal-lysosomal trafficking and protein sorting during the mid-phase of differentiation. This spike might be necessary to support the initial stages of cellular reorganization and membrane trafficking required for differentiation but stabilizes once these processes are well-established. The coordinated expression of *PINK1* and *PARK2* suggested that mitophagy and mitochondrial quality control played a crucial role in the differentiation process, likely supporting metabolic demands during neurogenesis. When analyzed as a whole, the patterns reflected a complex process of neurogenic differentiation in SH-SY5Y cells, with early dopaminergic features and later structural and maturation changes.

The proteomic analysis by nLC-MS/MS provided a broader view of the differentiation process. Some proteins were consistently regulated at all three time points, which suggested that these proteins might play crucial roles in the overall differentiation process. The proteins unique to each day point are likely to represent the proteins that were specifically important for distinct phases of differentiation. For example, differentially regulated proteins present only at the day 3 time point could be involved in the initiation of differentiation and might regulate early signaling pathways and morphological changes. In contrast, proteins present only on the day 6 time point were likely to play roles in mid-differentiation events and might involve in maturation processes or metabolic shifts in the differentiating cells. Proteins present only at a later stage could be involved in the maintenance or finalization of the neuronal phenotype.

The heatmap analyses revealed that the differentiation process had a significant impact on the cellular proteome, including the cell differentiation process (Figures 4A and 4B). According to the STRING analyses, the differentiation-related clusters, such as epithelial cell differentiation, keratinocyte differentiation, and neurogenesis, indicated intrinsic plasticity and heterogeneity of SH-SY5Y cells (Figure 4C). Neural progenitor cells exhibit epithelial characteristics in their early development state. However, during neuronal differentiation, some epithelial features are gradually lost (Heiman, 2022). Therefore, it is not unexpected that proteins related to epithelial cell differentiation are prominent in STRING analyses. Although neuronal differentiation markers are affected, they did not emerge as dominant over other pathways, which suggests the differentiation is initiated but not fully completed toward a neuronal fate within the given regimen. Quantification of expression patterns of neurogenesis-associated proteins throughout the differentiation period indicates a gradual increase in proteins like TUBA1A, ACTG1, and DBN1 that reflect their pivotal role in cytoskeletal remodeling and neuronal morphogenesis. However, the asynchronous expression patterns observed across markers further reinforced the notion of heterogeneity in the differentiation process. Figure 4D specifically focused on proteins upregulated during neurogenesis, highlighting their temporal dynamics in this in vitro system. The upregulation of BASP1, SCG2, and VGF aligns with their known roles in neuronal differentiation and synaptic function, lending further evidence to the dopaminergic characteristics acquired by the SH-SY5Y cells (Figure 4E). However, the incomplete or transient expression profiles suggested that these cells might not achieve full neuronal maturity without further optimization of differentiation protocols.

Overall, the intricate interplay between differentiation pathways and neurogenesis underscored both the potential use of differentiated SH-SY5Y cells as an in vitro PD model and its limitations. Future studies might focus on minimizing heterogeneity by refining differentiation protocols or exploring coculture systems to enhance functional maturity and stability. Additionally, to support the literature, the identified proteins need to be confirmed by Western blotting and two-dimensional gel electrophoresis (2DE).

## Supplementary Information







## Footnotes

1 Heatmapper (2025). expression [online]. Website: http://www.heatmapper.ca/expression [accessed 20 February 2025]

2 GO (2025). QuickGO annotations [online]. Webside: https://geneontology.org/ (release date 22.07.2025) [accessed 25 July 2025]

3 UniProt (2025). proteomes [online]. Webside: https://www.uniprot.org/ (release date 24.07.2024) [accessed 05 March 2025]

4 STRING (2025). network [online]. Webside: https://string-db.org/ (version 12.0) [accessed 11 November 2024]

5 HPA (2025). humanproteome/brain [online]. Website: https://www.proteinatlas.org/ Version: 24.0 [accessed 02 March 2025]

6 Geneglobe (2020). Analyze [online]. Website: https://geneglobe.qiagen.com/us/analyze [accessed 02 March 2020]
