# Supplementary material for: Evaluating SH-SY5Y cells as a dopaminergic neuronal model: morphological, transcriptomic, and proteomic insights
Source: Turk J Biol. 2025 Aug 11;49(6):700–11. doi: 10.55730/1300-0152.2772 (PMC12604937; doi:10.55730/1300-0152.2772)
Supplement: Supplementary file 1 [file Supplementary_File_1.docx]

**Supplementary File 1**

**Chemicals**

- 50 bp DNA Ladder (Invitrogen, US, 10416014)
- Agarose (Molecular Biology Grade) (Thermo Scientific, US, 17850)
- B-27™ Supplement (50X), serum free (Gibco, US, 17504044)
- DAPI and Hoechst Nucleic Acid Stains (Thermo Scientific, US, 62248)
- DMEM 1X with 4.5 g/L Glucose & L-Glutamine w/o Sodium Pyruvate 500 ml (Wisent Inc., QC, 319-015 CL)
- Ethidium Bromide (Sigma-Aldrich, US, E1510)
- Fetal Bovine Serum (FBS), Heat Inactivated, Collected in South America (Capricorn Scientific, DE, FBS-HI-12A)
- Fetal Bovine Serum Advanced (FBS Advanced), Collected in South America (Capricorn Scientific, DE, FBS-11B)
- Formaldehyde solution (Sigma-Aldrich, US, F8775)
- Formic Acid (Merck, US, 543804)
- GlutaMAX™ Supplement (Gibco, US, 35050061)
- Human Brain-Derived Neurotrophic Factor (BDNF) Recombinant Protein (Cell Signaling Technology, US, 3897)
- In-solution tryptic digestion and guanidination kit (Thermo Fisher Scientific, US, 89895).
- L-Glutamine (200 mM) (Biochrom, UK, K 0282)
- LTQ Velos ESI Positive Ion Calibration Solution (Thermo Scientific, US, 88323)
- MaxGel™ Extracellular matrix (Sigma-Aldrich, US, E0282)
- Mowiol (Sigma-Aldrich, US, 81382)
- N6,2′-O-Dibutyryladenosine 3′,5′-cyclic monophosphate sodium salt (Sigma-Aldrich, US, D0627)
- Neurobasal™ Medium (Gibco, US, 21103049)
- Penicillin/Streptomycin 10.000 U/10.000 µg/ml (Biochrom, UK, A 2212)
- Phosphate Buffered Saline (Sigma-Aldrich, US, 806552)
- Qubit Assay Kit (Invitrogen, US, Q33211)
- Quick Start™ Bradford Protein Assay Kit 1 (BioRad, US, 5000201)
- Retinoic acid, all trans (Enzo Life Sciences, US, BML-GR100)
- RevertAid First Strand cDNA Synthesis Kit (Thermo Scientific, US, K1621)
- RNeasy Mini Kit (50) (Qiagen, US, 74104)
- SYBR™ Green Universal Master Mix (Applied Biosystems™, US, 4344463)
- Triton™ X-100 (Sigma-Aldrich, US, X100)
- Unstained Protein MW Marker (Thermo Scientific, US, 26610)
- VLE DMEM (very low endotoxin with 4,5 g/l Glucose, with 3,7 g/l NaHCO_3_, with Na-Pyruvate, with stab. glut.) (Biochrom, UK, FG 1445)
