# Supplementary material for: Evaluating SH-SY5Y cells as a dopaminergic neuronal model: morphological, transcriptomic, and proteomic insights
Source: Turk J Biol. 2025 Aug 11;49(6):700–11. doi: 10.55730/1300-0152.2772 (PMC12604937; doi:10.55730/1300-0152.2772)
Supplement: Supplementary file 2 [file Supplementary_File_2.docx]

| **Antibodies** | **Catalog No** | **Dilution** |
| --- | --- | --- |
| Nestin (Primary Antibody) | Novus Biologicals, CO, NB300-266 | 1:50 |
| NeuroD1 (Primary Antibody) | Novus Biologicals, CO, H00004760-M01 | 1:100 |
| NeuN (Primary Antibody) | Novus Biologicals, CO,  NBP1-92693 | 1:500 |
| Nurr1 (Primary Antibody) | ThermoFisher Scientific, US, #MA1-195 | 1:500 |
| TH (Primary Antibody) | Novus Biologicals, CO, NB300-109 and ThermoFisher Scientific, US, 701949 | 1:1000, and  1:250 |
| Texas Red Anti Mouse (Secondary Antibody) | Thermo Scientific, US, T-6390 | 1:1000 |
| Texas Red Anti Rabbit (Secondary Antibody) | Thermo Scientific, US, T-6391 | 1:1000 |

**Table 1.** Primary and secondary antibody information for neuronal differentiation.

| **Gene Name** | **Gene Symbol** | **Refseq No.** | **Catalogue ID.** | **Expected Band ​** |
| --- | --- | --- | --- | --- |
| Nestin | NES | NM_006617 | PPH02388A | 161 |
| Neuronal Differentiation 1 | NEUROD1 | NM_002500 | PPH00039E | 110 |
| RNA Binding Fox-1 Homolog 3 | NeuN/ RBFOX3 | NM_001082575 | PPH21523B | 139 |
| Microtubule Associated Protein 2 | MAP2 | NM_002374 | PPH02419A | 172 |
| Nuclear Receptor Subfamily 4 Group A Member 2 | Nurr1/ NR4A2 | NM_006186 | PPH02082A | 164 |
| Tyrosine Hydroxylase | TH | NM_000360 | PPH02062E | 54 |
| Sodium-Dependent Dopamine Transporter | DAT/ SLC6A3 | NM_001044 | PPH01449A | 153 |
| Aromatic l-amino acid decarboxylase | DDC | NM_000790 | PPH19374B | 62 |
| Dopamine Receptor D1 | DRD1 | NM_000794 | PPH01857F | 107 |
| PTEN Induced Kinase 1 | PINK1 | NM_032409 | PPH20890B | 136 |
| Parkin RBR E3 Ubiquitin Protein Ligase | PARK2 | NM_004562 | PPH05959C | 108 |
| Parkinsonism Associated Deglycase | DJ-1/PARK7 | NM_007262 | PPH19854F | 102 |
| VPS35 Retromer Complex Component | VPS35 | NM_018206 | PPH22095A | 149 |
| Actin Beta | ACTB | NM_001101 | PPH00073G | 174 |
| Glyceraldehyde-3-Phosphate Dehydrogenase | GAPDH | NM_002046 | PPH00150F | 130 |

**Table 2.** Detailed information on the genes monitored during neurogenic differentiation of SH-SY5Y cells.
